# The SPACES Framework: Sustainable Oral Health Programs for Older Adults

**DOI:** 10.3390/dj14070433

**Published:** 2026-07-13

**Authors:** Joanna Cheuk Yan Hui, Lindsey Lingxi Hu, Ivy Gaofang Sun, Alice Kit Ying Chan, Chun Hung Chu

**Affiliations:** Faculty of Dentistry, The University of Hong Kong, Hong Kong 999077, China; cyjaonna@hku.hk (J.C.Y.H.); hu_lingxi@connect.hku.hk (L.L.H.); ivysun24@hku.hk (I.G.S.); dralice@hku.hk (A.K.Y.C.)

**Keywords:** oral health, older adults, healthy aging, community oral health programs, health equity, implementation framework, integrated care

## Abstract

**Background/Objectives:** Traditional community oral health programs (COHPs) often remain reactive, clinic-based, and insufficiently integrated with medical, social, and aged-care systems. As a result, they may be poorly positioned to address the needs of older adults experiencing multimorbidity, mobility limitations, cognitive impairment, care dependency, and financial or geographic barriers. This paper proposes SPACES, an implementation-oriented conceptual framework for designing, delivering, and evaluating community oral health programs for older adults. **Methods:** SPACES was developed through a targeted interpretive synthesis of peer-reviewed literature, policy guidance, and implementation-oriented sources related to oral health, healthy aging, integrated care, access barriers, workforce development, and sustainable service delivery. **Results:** The framework comprises six interconnected domains: Scalable Prevention and Education, Partnerships Across Sectors, Adaptive Care Coordination, Community Access Solutions, Empowered Workforce Development, and Sustainable Service Models. It is intended primarily for adults aged 60 years and older, while allowing adaptation in jurisdictions that use 65 years and older as the eligibility threshold. The framework applies across community, home care, assisted living, and long-term care settings. SPACES organizes evidence-informed components for older-adult oral health, including routine screening, caregiver-supported daily mouth care, risk-stratified prevention, referral pathways, proximity-based service delivery, geriatric workforce competencies, task sharing, sustainable financing, governance, and quality improvement. **Conclusions:** SPACES is presented not as a tested intervention but as a conceptual and program-planning tool to help planners clarify responsibilities, identify equity gaps, align cross-sector resources, and select measurable implementation indicators. Future stakeholder refinement and empirical evaluation are needed through co-design, pragmatic implementation studies, and real-world assessment of feasibility, acceptability, cost, equity impact, and oral health outcomes.

## 1. Introduction

Oral health is a fundamental yet often neglected component of holistic well-being and healthy aging. This neglect is especially consequential in the context of global population aging. By 2050, the global population aged 60 years and older is projected to reach 2.1 billion, with 80% living in low- and middle-income countries where many health systems face substantial resource constraints [1]. This demographic change is occurring alongside a substantial global burden of preventable oral disease. The World Health Organization (WHO) estimates that almost half the world’s population has an oral disease. Untreated dental caries affects approximately 2.5 billion people, and severe periodontal disease affects about 1 billion people, contributing to a large preventable disease burden across the life course [2]. Recent WHO policy developments have further emphasized the need to integrate oral health into universal health coverage, noncommunicable disease prevention, primary care, workforce planning, financing, and health-system monitoring [3]. The consequences extend beyond physical health, causing oral pain, tooth loss, and disfigurement, which can lead to social isolation, depression, and reduced nutritional intake, undermining healthy aging [4,5]. However, oral health remains marginalized in geriatric care, constrained by fragmented health systems, ageist assumptions, and limited policy attention.

Older adults experience multiple, intersecting barriers to maintaining oral health and accessing care. Physiological changes associated with aging can increase susceptibility to disease; for example, reduced salivary flow, often related to medication use, elevates the risk of dental caries and oral infections [6]. Cognitive impairment, including dementia, can further compromise daily self-care, making routine practices such as toothbrushing inconsistent or dependent on caregiver support [7]. Socioeconomic factors also intensify these risks: limited disposable income and high out-of-pocket dental costs can force trade-offs between oral health care and essentials such as food, housing, or medications [8]. Additionally, mobility limitations, transportation barriers, and care dependency can isolate rural, homebound, and institutionalized older adults from clinic-based services. Cultural beliefs and organizational factors further shape oral health outcomes. One persistent misconception, held by some patients and providers, is that tooth loss is an inevitable part of aging. Such beliefs can normalize symptoms, delay preventive or restorative care, and reduce expectations for oral health in later life. Studies in long-term care settings have reported limited access to routine dental care, low uptake of available services, and gaps in staff training to identify and respond to oral health problems [9,10]. These gaps reflect not only clinical shortcomings but also a broader failure to realize the WHO vision of healthy aging, which emphasizes functional ability and dignity throughout later life.

Existing community oral health programs (COHPs) often struggle to address these layered risks and access barriers. In many settings, services remain reactive, prioritizing urgent, episodic treatment rather than prevention, continuity, and coordinated care. This pattern can perpetuate cycles of pain, repeated emergency service use, and avoidable costs. One visible downstream consequence is reliance on urgent and emergency services for dental problems that are rarely definitively treated. In the United States, Healthcare Cost and Utilization Project (HCUP) estimates suggest that treat-and-release emergency department visits for dental conditions among adults aged 65 years and older cost approximately USD 211 million in 2019, although such care is typically symptom-focused rather than definitive [11,12]. In Australia, potentially preventable hospitalizations for dental conditions are used as an indicator of unmet need and access barriers, particularly among disadvantaged groups [13]. Workforce constraints compound these challenges. Formal recognition of gerodontology and associated training pathways varies by jurisdiction [14,15]. Moreover, oral health training in primary care remains limited, reducing opportunities for prevention, early detection, and coordinated oral–systemic management [16]. Even when oral health services are available, they are often not systematically integrated with medical care, social support, home care, or aged-care systems. These disconnects reflect a broader undervaluation of oral health in aging and highlight the need for an implementation-oriented model that can support prevention, integration, accessibility, workforce development, and sustainability.

In response to this need, this article proposes SPACES as a conceptual framework to strengthen COHPs for older adults. SPACES comprises six interconnected domains: Scalable Prevention and Education, Partnerships Across Sectors, Adaptive Care Coordination, Community Access Solutions, Empowered Workforce Development, and Sustainable Service Models. This article does not report original empirical data, a formal systematic review, or an empirically validated framework-development study. The aim of this article is therefore to clarify what the framework is, how it was conceptually derived, where it may add value, and what requires further empirical testing and stakeholder validation.

## 2. Framework Development Approach

### 2.1. Design and Purpose

SPACES was developed as a conceptual and implementation-oriented framework. Its purpose was to synthesize recurring service functions and implementation enablers relevant to community oral health programs for older adults and to organize them into a practical framework for program planning, adaptation, and evaluation.

### 2.2. Source Identification

The framework was informed by an interpretive synthesis of peer-reviewed literature, policy documents, practice guidance, and implementation-oriented sources. Sources were identified through purposive searches of PubMed/MEDLINE, Google Scholar, and organizational websites, including the World Health Organization, FDI World Dental Federation, Australian Institute of Health and Welfare, National Institute for Health and Care Excellence, Agency for Healthcare Research and Quality, National Academies, OECD, and professional or regulatory bodies. Searches were conducted up to 30 June 2026. Search concepts combined terms related to older adults, aging, geriatric dentistry, oral health, dental caries, periodontal disease, xerostomia, dentures, long-term care, home care, dementia, primary care integration, community oral health, care coordination, referral pathways, teledentistry, mobile dental services, transportation, affordability, task-sharing, workforce development, financing, governance, sustainability, and equity. This was a purposive, non-exhaustive synthesis; no PRISMA-type screening flow or formal quality appraisal was intended.

### 2.3. Eligibility and Source Selection

Sources were considered relevant if they addressed at least one of the following: (1) older adults’ oral health needs; (2) prevention or early detection of oral disease; (3) oral health in long-term care, home care, or community settings; (4) integration of oral health into primary or social care; (5) access barriers (e.g., cost, mobility, geography, culture, language, or disability); (6) caregiver-supported oral care; (7) teledentistry, mobile dental services, or other outreach models; (8) workforce development, task-sharing, or community health workers; or (9) financing, governance, sustainability, implementation, and quality improvement.

### 2.4. Interpretive Synthesis and Domain Derivation

The synthesis used an interpretive, framework-building approach. Concepts were organized based on the oral health problem, target population and setting, service function, implementation needs, equity implications, and monitoring indicators. These were grouped into candidate domains through iterative comparison. Domains were retained if relevant to older adults, actionable, distinct, and linked to equity, feasibility, or sustainability. Six SPACES domains emerged as key program levers. Conceptual sufficiency was achieved when new sources aligned with these domains without adding new service functions or implementation strategies. Table 1 summarizes the main design questions and contributions of each domain.

### 2.5. Use of WHO Guidance

WHO documents were used as normative anchors for SPACES. The framework was aligned with WHO priorities on healthy aging, universal health coverage, integration of oral health into broader health systems, prevention-oriented care, workforce strengthening, and monitoring of oral health inequalities [17]. These principles informed the framework’s equity orientation and emphasis on prevention, access, coordination, workforce capacity, financing, governance, and quality improvement. The WHO global oral health monitoring framework report also informed the emphasis on measurable indicators, equity-sensitive monitoring, and alignment with global oral health targets toward 2030 [3].

### 2.6. Stakeholder Involvement

No formal stakeholder co-design, Delphi process, expert panel, or patient/public involvement exercise was conducted during the initial development of SPACES. Future refinement should include older adults, caregivers, dental professionals, primary care teams, aged-care staff, community organizations, policymakers, and payers to assess relevance, feasibility, acceptability, cultural safety, workload, and implementation burden.

## 3. SPACES Framework: Overview and Scope

**Figure 1:**
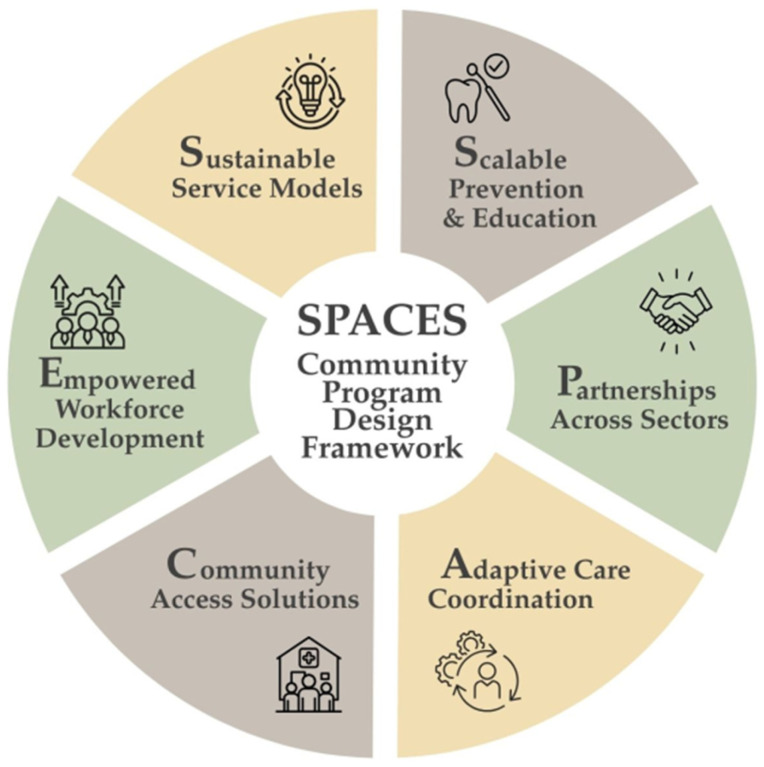
The six interrelated domains of the SPACES framework (created with Canva, accessed on 30 April 2026).

SPACES provides an organizing structure for identifying program gaps, assigning responsibilities, selecting feasible service-delivery strategies, and monitoring equity-sensitive implementation indicators. In this framework, “older adults” generally refers to adults aged 60 years and above, consistent with WHO aging statistics, while recognizing that many health systems define older-adult service eligibility using 65 years and above. SPACES is intended to be adapted according to local population definitions, eligibility rules, workforce regulations, financing arrangements, and service capacity. The six domains in SPACES are interdependent but operationally distinct. Scalable Prevention & Education defines what preventive and educational activities should be embedded into routine care. Partnerships Across Sectors defines which organizations and sectors must collaborate to provide oral health support. Adaptive Care Coordination defines how individual care plans are reassessed and adjusted as risks change. Community Access Solutions defines how services become physically, financially, culturally, and administratively reachable. Empowered Workforce Development defines who delivers care and what competencies, supervision arrangements, and role clarity are required. Sustainable Service Models define how programs are financed, governed, monitored, and maintained over time (Figure 1).

The added value of SPACES is not that each individual activity is new. Many components, including oral health screening, fluoride-based prevention, caregiver training, teledentistry, task-sharing, mobile services, and referral pathways, already appear in gerodontology, public health dentistry, integrated care, and WHO policy literature [18]. The contribution of SPACES is to organize these activities into a single, older-adult-focused program design framework that links service functions to implementation enablers and equity monitoring across community, home care, and long-term care settings [2,3,19].

### 3.1. Scalable Prevention & Education

Scalable Prevention & Education shifts COHPs for older adults from reactive, episodic treatment toward proactive prevention embedded in routine care. This domain emphasizes brief, repeatable activities delivered in the settings where older adults live and receive support, including primary care, home care, long-term care, and community settings. Core activities include early risk identification, daily mouth care support, practical education, and risk-stratified professional prevention for individuals at higher risk. The aim is to reduce avoidable pain, infection, caries progression, and functional decline. Recent reviews of oral health and healthy aging have highlighted the need for better integration of oral health into geriatric care and have identified common intervention components, including education, practical demonstrations, caregiver support, and topical fluoride-based prevention [20].

Older adults face vulnerabilities that make prevention particularly important. Age-related physiological changes, including thinning of the oral mucosa and reduced salivary flow, may increase susceptibility to dental caries, mucosal disease, and oral infections [21]. Chronic conditions such as diabetes, arthritis, and cognitive impairment can also limit oral hygiene capacity, while commonly prescribed medications for hypertension, depression, and other conditions may contribute to xerostomia and accelerate tooth decay [22,23]. These factors can interact in reinforcing cycles: oral pain and tooth loss may impair diet quality and social participation, while poor oral health can complicate chronic disease management.

*Brief screening and escalation pathways:* Programs should include a brief, standardized oral health screening within existing workflows such as long-term care admission, home care assessments, or chronic disease reviews. Tools like the Oral Health Assessment Tool (OHAT) or a locally validated equivalent can help ensure consistent documentation and action [24,25]. Screening results should lead to an individualized prevention plan with review intervals, explicit escalation criteria, and designated responsibility for follow-up.

*Practical education and self-management support:* Education should be brief, practical, culturally appropriate, and responsive to health literacy and cognitive needs. Strategies may include demonstrations, teach-back methods, visual prompts, and caregiver-inclusive messaging [26,27]. Key content includes daily oral care routines, denture hygiene, dietary advice that balances nutrition with caries risk, tobacco-cessation support, and recognizing symptoms that require care. Importantly, education must be combined with enabling conditions, such as access to supplies and clear referral pathways; otherwise, knowledge alone may not lead to lasting behavior change.

*Teledentistry-supported early detection and follow-up:* When feasible, teledentistry can support preventive workflows by enabling remote triage, caregiver coaching, review of oral hygiene or healing, and prioritization of in-person visits for homebound or geographically isolated older adults [28,29]. It should support, rather than replace, clinical pathways and should include clear procedures for consent, privacy, documentation, and escalation to face-to-face care.

*Risk-stratified preventive care:* For older adults at elevated caries risk, particularly those with exposed root surfaces or reduced salivary flow, evidence supports considering fluoride-based prevention and minimally invasive approaches such as high-fluoride toothpaste, fluoride varnish, and silver diamine fluoride where clinically appropriate and permitted by local regulation [18].

*Supported daily mouth care and caregiver coaching:* Family members and professional caregivers are often the first line of support for dependent older adults. Programs should include practical coaching in safe oral care techniques, denture cleaning, managing resistance in dementia, recognizing nonverbal pain cues, and basic infection control practices [30]. This component focuses on daily prevention at the point of care, while broader workforce training and supervision are addressed under Empowered Workforce Development.

### 3.2. Partnerships Across Sectors

Partnerships Across Sectors addresses inter-organizational collaboration and governance rather than individual-level care coordination. This domain responds to service fragmentation by aligning oral health services with primary care, social services, long-term care providers, community organizations, transportation services, nutrition programs, and policymakers. It serves as the system-level coordination backbone of the framework, helping ensure that prevention, early detection, and treatment are supported by practical conditions such as transportation, caregiving support, accessibility accommodations, nutrition support, and affordability [3,31].

*Shared entry points and accountable referral pathways:* Effective partnerships create oral health entry points within services that older adults already use, including primary care visits, home care assessments, long-term care reviews, pharmacy encounters, and community senior services [32]. At the partnership level, the emphasis is on agreed referral pathways, role definitions, documentation expectations, and feedback loops across organizations. These arrangements help ensure that oral health concerns identified in non-dental settings are not simply recorded but acted upon [33].

*Enabling support through cross-sector collaboration:* Partnerships are most useful when they connect clinical prevention with the social and functional supports needed to make care possible. These may include transportation coordination, appointment navigation, reminder systems, nutrition support, caregiver assistance, and accessibility accommodations [34,35]. In this domain, the focus is not on the details of each access intervention but on how organizations collaborate to make those supports available.

*Shared training and role agreements:* Cross-sector partnerships can support common training expectations, escalation procedures, and supervision arrangements across dental, medical, community, and long-term care settings. This helps clarify which oral health activities non-dental personnel can perform, when professional dental input is required, and how to escalate concerns [35]. The specific competencies required for safe delivery are addressed under Empowered Workforce Development.

*Governance, policy alignment, and data sharing:* Sustained collaboration requires governance arrangements that extend beyond short-term projects. These include clear role definitions, shared performance indicators, data-sharing agreements, referral accountability, and policy or financing mechanisms that support prevention and referral completion. [3,33]. This domain is distinct from Sustainable Service Models. Partnerships Across Sectors focuses on how multiple organizations collaborate, whereas Sustainable Service Models focuses on the program’s long-term operating model.

### 3.3. Adaptive Care Coordination

Adaptive Care Coordination focuses on the individual older adult’s care journey over time. It ensures that oral health support remains continuous, timely, and responsive as health status, functional ability, living arrangements, and care needs change. Unlike Partnerships Across Sectors, which address system-level relationships, Adaptive Care Coordination addresses person-level reassessment, handoffs, escalation, and follow-up [36,37,38].

*Dynamic reassessment with actionable triggers:* Oral health needs should be reassessed when there are changes in medical status, medication use, functional ability, cognition, diet, or living situation. Practical triggers may include reduced self-care capacity, new swallowing difficulties, increased dependence on hygiene assistance, new oral symptoms, xerogenic medications, chemotherapy, worsening diabetes control, dehydration, or major dietary changes [39,40]. Reassessment should lead to concrete actions, such as changes to the prevention plan, caregiver support, referral urgency, or follow-up interval.

*Transitions and handoffs:* Older adults frequently move among home, hospitals, rehabilitation, assisted living, and long-term care settings. Standardized handoffs can reduce missed oral health needs by including prompts for oral pain, chewing or swallowing ability, denture status, mouth-care capacity, required supplies, recent screenings, and pending dental follow-up [37,41]. These prompts help oral health remain visible during transitions that often prioritize acute medical concerns [33].

*Information flows and follow-through:* Effective coordination requires reliable information flow and clear accountability. Useful features may include alerts for xerostomia risk, overdue oral health screening, shared care plans, referral tracking, and teledentistry-supported triage for homebound individuals [29]. However, high-technology systems are not essential in all settings. The minimum requirement is a reliable process for recording concerns, communicating them to the appropriate provider, and confirming that follow-up occurred [42].

*Flexible resourcing and role clarity:* Programs should define who coordinates care, who monitors follow-up, who contacts the older adult or caregiver when appointments are missed, and which urgent support can be mobilized quickly. The central question in this domain is not “Which organizations are linked?” but rather “How does this individual older adult move safely and reliably through care?” [35].

### 3.4. Community Access Solutions

Community Access Solutions addresses the geographic, financial, administrative, and cultural barriers that prevent older adults from receiving timely oral health care [43,44]. Access is a design issue: even well-designed services will have limited impact if older adults cannot physically, financially, administratively, or culturally reach them. This domain focuses on the user-facing experience of access.

*Proximity-based delivery models:* COHPs can expand reach through mobile dental units, pop-up services in community centers, and on-site services in long-term care and assisted living facilities [45]. These models are particularly relevant for older adults who are homebound, have limited transportation, or live in underserved areas [46].

*Transportation and navigation support:* Access can be improved through community transport, appointment accompaniment, reminder systems, simplified booking processes, and navigation assistance [47,48]. These supports are especially important for older adults with mobility limitations, cognitive impairment, low health literacy, limited digital access, or fragmented care arrangements [49].

*Affordability mechanisms and administrative simplicity:* Sliding-scale fees, subsidized preventive packages, and voucher systems can improve access, but these mechanisms must be easy to use and low-burden administratively [50]. Complex forms, unclear eligibility rules, or repeated documentation requirements may discourage participation even when financial support is available.

*Cultural safety, language access, and trusted community entry points:* Programs can improve uptake by partnering with trusted institutions and ensuring language-concordant communication, interpretation, continuity of staff where possible, and culturally safe care [51,52,53]. This is especially important for older adults from minoritized, immigrant, Indigenous, rural, or socially isolated communities. This domain is distinct from Partnerships Across Sectors because it focuses on the barriers older adults and caregivers face when trying to use services. It is also distinct from Sustainable Service Models, which focus on the program’s back-end viability rather than the user-facing access experience.

### 3.5. Empowered Workforce Development

Empowered Workforce Development equips the workforce delivering COHPs, including dental teams, primary care partners, aged-care staff, community health workers, and caregivers [2,43]. Access and coordination can only translate into improved outcomes when the workforce is prepared to deliver care that is geriatric-appropriate, culturally safe, feasible in community settings, and responsive to functional and cognitive limitations [4].

*Geriatric-specific clinical and communication competence*: Priority competencies include dementia-capable communication, supported decision-making, management of xerostomia and polypharmacy-related risk, prevention and management of root caries, denture assessment, and oral care in serious illness or palliative contexts [54]. Training should also address respectful communication, trauma-informed practice, and culturally safe engagement with older adults and caregivers.

*Interdisciplinary role design and task-sharing:* Task-sharing can extend program capacity when activities are matched to the lowest safe and competent provider level [55]. For example, non-dental personnel may support screening, daily mouth care, education, and referral navigation, while dental professionals retain responsibility for diagnosis, complex prevention planning, and treatment. Feasibility depends on local scope-of-practice regulation, supervision structures, liability arrangements, and competency assurance [4,56]. Recent workforce literature emphasizes that task-sharing depends not only on training but also on regulatory scope, supervision, remuneration, workplace organization, referral systems, and the acceptability of expanded roles to patients and professionals [55].

*Point-of-care support and continuous learning:* Workforce development should not rely on one-time training alone. Decision aids, mentoring, refresher training, case conferences, audit-and-feedback, teleconsultation, and onboarding support can improve consistency and confidence [57,58,59]. These supports are particularly important in long-term care and home care settings, where staff turnover and competing care priorities can undermine implementation. Evidence from community nursing and home care settings suggests that oral-health responsibilities are more likely to be sustained when staff receive practical training, workflow support, professional supervision, and implementation resources rather than one-off education alone [60].

*Workforce sustainability and retention:* Older-adult oral health care can be time-intensive, physically demanding, and emotionally challenging. Programs should consider caseloads, travel time, safety procedures for home visits, supervision, psychological support, and career development opportunities [61,62,63]. Supporting the workforce is essential for maintaining quality, continuity, and morale over time.

### 3.6. Sustainable Service Models

Sustainable Service Models addresses the long-term operating model of community oral health programs. This domain focuses on how programs remain dependable, scalable, and affordable beyond short-term pilots or grant cycles. Sustainability depends on stable financing, appropriate staffing, governance, supply chains, data systems, and continuous quality improvement [3,64].

*Staffing models and role optimization:* Sustainable programs match tasks to the appropriate level of training, supervision, and risk. This may include task shifting or task sharing where legally and clinically appropriate, but sustainability also requires stable staffing patterns, backup coverage, supervision time, and realistic workload expectations [63,64,65]. This domain builds on Empowered Workforce Development by translating competencies into a durable staffing model.

*Digital infrastructure and workflow efficiency:* At the program level, digital tools may support scheduling, referral tracking, follow-up reminders, stock monitoring, quality-improvement cycles, and performance reporting. However, technology should be selected according to local capacity and should not create new inequities for older adults with limited digital access, sensory impairment, cognitive impairment, or language barriers. Digital systems are most useful when they simplify workflows, strengthen accountability, and reduce administrative burden rather than adding complexity [58].

*Stable financing and aligned incentives:* Sustainable models depend on predictable funding that supports prevention, outreach, and coordination rather than episodic treatment alone [66,67]. Financing arrangements should account for the additional time required by older adults with mobility limitations, cognitive impairment, multimorbidity, or complex social needs. Incentives should support prevention, equity, and continuity rather than volume alone.

*Governance, quality improvement, and supply-chain reliability*: Programs should monitor coverage, wait times, referral completion, preventive service delivery, patient experience, and equity indicators. Quality improvement processes can help identify gaps in access, follow-up, workforce capacity, and outcomes [68,69]. Sustainable programs also require reliable supply chains for preventive materials, infection-control supplies, portable equipment, and denture-care resources, as well as contingency plans for staff shortages, service disruptions, and changing community needs.

## 4. Applying SPACES in Practice

### 4.1. Implementation Principles and Practical Sequence

**Figure 2:**
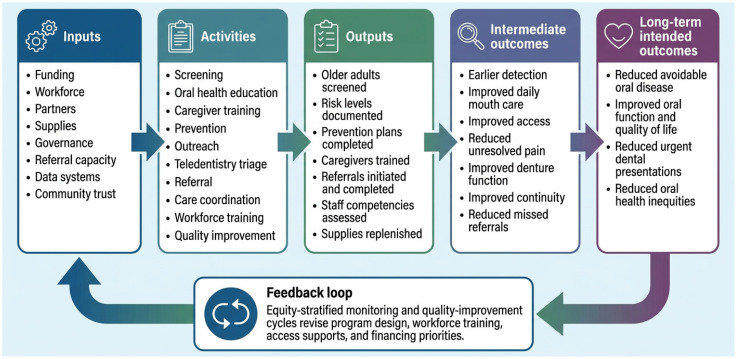
Proposed implementation logic model for the SPACES framework (created with Canva on 20 June 2026).

Implementation of SPACES should be staged rather than attempted as a single all-at-once reform. Programs should begin with minimum viable components, such as routine screening, basic prevention workflows, caregiver education, and closed-loop referral pathways, before expanding to mobile services, teledentistry, advanced data systems, or new financing models. The sequence and intensity of implementation should reflect local workforce regulations, funding, treatment capacity, digital infrastructure, transportation options, and the needs of the older-adult population served. Figure 2 presents a proposed implementation logic model linking program inputs, activities, outputs, intermediate outcomes, longer-term intended outcomes, and feedback for continuous improvement.

A practical implementation starts with identifying the target population and equity goals, then mapping local resources such as dental providers, primary care, aged-care services, transportation, and language access. It also involves understanding regulatory limits and benefit coverage. Next, governance is established by appointing a lead organization, defining roles, referral processes, data sharing, and quality improvement routines. Implementation focuses on creating screening and prevention workflows, selecting simple oral health screening tools, setting review intervals, and ensuring access to toothbrushes, fluoride toothpaste, dentures, and referral information. Clear pathways for urgent and routine referrals, response times, appointment support, transportation, and feedback mechanisms are crucial. Role-specific training for dental teams, primary care staff, aged-care workers, and caregivers is essential, along with competency checks and supervision. Finally, programs should monitor progress and quality using straightforward indicators that track service reach, referral completion, treatment access, oral health outcomes, caregiver burden, and disparities among vulnerable groups.

### 4.2. Adapting SPACES to Different Resource Settings

SPACES should be adapted according to resource level rather than implemented as a fixed package. In high-resource settings, programs may use electronic health records, teledentistry platforms, mobile dental units, dental therapists or hygienists, integrated scheduling systems, and value-based payment arrangements. In middle-resource settings, programs may rely on pop-up clinics, shared referral forms, community health workers, telephone follow-up, basic registries, and partnerships with local dental providers. In low-resource settings, programs may begin with paper-based screening, caregiver education, basic prevention kits, community-based referral agreements, scheduled outreach visits, and low-bandwidth communication such as telephone triage. Regardless of resource level, programs should avoid screening older adults without a realistic pathway for prevention, advice, urgent escalation, or treatment. Screening without referral capacity may increase unmet need, frustration, and ethical risk. Therefore, the minimum implementation should include at least one feasible pathway for urgent care, one pathway for routine dental referral, and one mechanism for caregiver or staff support. Selected SPACES activities vary in their evidence base, implementation requirements, and potential risks; these considerations are summarized in Table 2.

### 4.3. Minimum Viable Components and Capacity Growth

Programs should assign clear responsibility for each implementation function, including governance, population identification, screening, prevention planning, referral and escalation, access support, workforce development, and quality improvement. Example responsible actors may include a program lead, dental service representative, aged-care supervisor, community partner, navigator, and quality-improvement lead. Table 3 summarizes minimum viable components, operational deliverables, and capacity-growth features. Equity-focused use of these indicators is discussed in Section 4.4.

### 4.4. Equity and Quality Monitoring

Since equity is central to SPACES, programs should clearly identify which inequalities they aim to reduce and how to measure progress. Oral health equity involves lowering unfair, avoidable differences in disease, access, oral function, pain, and treatment among older adults. Monitoring should cover the entire care pathway, not just entry points. Programs can assign responsible actors and track key indicators like screening, prevention, referral completion, urgent assessment wait times, training, reach among vulnerable groups, acceptability, and resource use. Where feasible, indicators should be stratified by key vulnerability characteristics (e.g., income/financial hardship, rurality, long-term care residence, cognitive impairment, disability, language barriers, digital exclusion, care dependency, and homebound status). Stratification helps ensure efforts reach those at greatest risk rather than mainly benefiting already connected individuals [17].

## 5. Relationship to Existing Frameworks and Policy Approaches

SPACES aligns with healthy aging, universal health coverage, oral health policy, integrated care, workforce, and sustainability approaches. Its added value lies in translating these broad principles into operational domains for community oral health program design for older adults. Table 4 summarizes how SPACES incorporates and extends existing frameworks and policy approaches [3].

## 6. Limitations and Future Research

SPACES is a preliminary conceptual and implementation-oriented framework rather than a validated intervention package. It does not provide empirical evidence that the use of SPACES improves oral health outcomes, reduces costs, or reduces inequalities. The proposed pathways are therefore hypothetical and require testing through pragmatic implementation studies, feasibility studies, process evaluations, cost analyses, and equity-focused outcome evaluations. The framework was developed through a targeted interpretive synthesis rather than a systematic or scoping review. Consequently, source identification was purposive and may not have captured all relevant literature, especially non-English sources, unpublished local program evaluations, or evidence from low- and middle-income countries. No formal risk-of-bias assessment or evidence grading was conducted. The framework may therefore reflect publication bias, policy-source bias, and high-income-country bias.

Several domains overlap in practice. For example, closed-loop referrals depend on partnerships, care coordination, workforce roles, information systems, and governance. The framework attempts to clarify domain boundaries, but real-world implementation will require local interpretation and may require combining domains into integrated workflows. Implementation may also be constrained by workforce shortages, scope-of-practice regulations, limited treatment capacity, reimbursement gaps, digital exclusion, transportation barriers, caregiver burden, supply-chain interruptions, and weak data systems. Screening may identify needs that exceed available dental capacity, creating ethical and operational challenges. Task-sharing and caregiver-enabled prevention may improve reach but require training, supervision, time, and support to avoid shifting excessive responsibility onto non-dental staff or unpaid caregivers. Finally, SPACES is intentionally adaptable, but this flexibility may reduce standardization and make comparison across settings more difficult.

Future research should refine SPACES through stakeholder co-design with older adults, caregivers, dental professionals, primary care providers, aged-care staff, community organizations, policymakers, and payers. Studies should identify which components are essential, which are context-dependent, and which combinations are most feasible and effective in high-, middle-, and low-resource settings. Pragmatic implementation studies are also needed to assess feasibility, acceptability, cost, equity impact, and oral health outcomes across diverse resource settings.

## 7. Conclusions

SPACES offers a preliminary, equity-oriented conceptual framework for organizing community oral health programs for older adults. By linking prevention, partnerships, adaptive coordination, access solutions, workforce development, and sustainability, the framework may help planners identify service gaps, assign responsibilities, and develop measurable implementation strategies across community, home care, and long-term care settings. However, SPACES has not yet been empirically validated. Future work should refine the framework through stakeholder co-design and test its feasibility, acceptability, cost, equity impact, and oral health outcomes in real-world implementation studies.

## Figures and Tables

**Table 1:** Operational boundaries of the SPACES domains.

SPACES Domain	Primary Design Question	Main Contribution
**Scalable Prevention & Education**	What preventive and educational activities should be routinely delivered?	Screening, oral health education, caregiver-supported daily mouth care, risk-based prevention, and early detection.
**Partnerships Across** **Sectors**	Which organizations must collaborate to make care possible?	Formal roles, referral agreements, navigation supports, transportation links, benefit coordination, and shared governance across health, social, and aged-care sectors.
**Adaptive Care** **Coordination**	How is an individual’s oral health plan revised as needs change?	Reassessment triggers, handoffs, closed-loop communication, transition planning, and coordination across settings.
**Community Access** **Solutions**	How do services become reachable and acceptable?	Mobile, pop-up, on-site, transport-supported, low-cost, culturally safe, and language-accessible service options.
**Empowered Workforce** **Development**	Who delivers care and what competencies are required?	Geriatric oral health skills, dementia communication, task sharing, supervision, mentoring, and workforce retention supports.
**Sustainable Service** **Models**	How is the program maintained over time?	Financing, governance, procurement, quality improvement, supply chains, resilience planning, and integration into existing systems.

**Table 2:** Evidence and implementation considerations for selected SPACES activities.

Activity	Evidence/Support Base	Implementation Requirements	Key Limitations or Risks
**Standardized oral health screening**	Validated tools and long-term care oral assessment literature	Staff training, documentation, referral criteria, review intervals	Screening alone does not improve outcomes without follow-up capacity
**Fluoride-based** **prevention and root** **caries management**	Stronger evidence bases for caries prevention and management	Clinical protocols, scope-of-practice compliance, supply chain, risk assessment	Requires adherence, supervision, and clinical judgment
**Silver diamine fluoride or minimally invasive care**	Growing evidence for caries arrest in appropriate populations	Consent, training, clinical criteria, follow-up, explanation of staining	Acceptability, regulation, and protocols vary by jurisdiction
**Caregiver-supported daily mouth care**	Supportive evidence, especially in dependent older adults and dementia care	Training, supplies, supervision, time allocation, resistance-management skills	May increase caregiver workload if not resourced
**Teledentistry triage and follow-up**	Emerging evidence for improving access and triage	Consent, privacy, documentation, digital access, escalation pathway	Digital exclusion, diagnostic limits, reimbursement barriers
**Mobile, pop-up, or** **on-site dental services**	Implementation evidence supporting access improvement	Equipment, maintenance, staffing, scheduling, infection control, referral capacity	Higher operational cost and sustainability challenges
**Navigation and** **transportation support**	Evidence from broader health access literature	Navigators, transport partnerships, reminders, accompaniment options	Requires funding and coordination beyond dental services
**Task-sharing and** **expanded workforce roles**	Supported by workforce and health-system guidance	Regulatory approval, supervision, competency assessment, escalation rules	Risk of role confusion or quality variation without governance
**Stable financing and** **value-oriented payment**	Health policy and payment-model literature	Funding agreements, outcome indicators, administrative simplicity	Context-specific; effectiveness requires evaluation

**Table 3:** SPACES—minimum viable components, operational deliverables, and capacity growth (scale-up/maturity).

SPACES Pillar	Viable Implementation	Operational Deliverables	Responsible Actors	Example Indicators	Capacity Growth (Scale-Up/Maturity)
**Scalable** **Prevention &** **Education**	Embed brief screening in routine touchpoints; practical education; supplies + restock pathway	Screening tool + completion rate log; prevention plan template; caregiver handout; training record; supply list + restock SOP	Site oral-health champion; nurse/home-care supervisor; dental outreach lead	Coverage of screening; proportion with prevention plan; supply stock-out frequency	Risk-stratified prevention bundles (e.g., fluoride therapy); teledentistry for coaching/follow-up; xerostomia mitigation workflow
**Partnerships Across Sectors**	Identify core partners; define roles; shared referral pathway; closed-loop communication	Memorandum of understanding/role descriptions; pathway map; referral form + feedback loop template; partner directory	Program lead; partner liaison; primary care/aged-care focal points	Number of active partners; referral feedback return rate; frequency of governance meetings	Co-located/pop-up services in partner sites; braided supports (navigation/transport/supplies); joint governance meetings; shared protocols
**Adaptive Care** **Coordination**	Define reassessment triggers; assign coordinator; standard handoffs; escalation timelines	Trigger checklist; handoff/transition template; escalation criteria; coordinator workflow + contacts	Care coordinator; nurse case manager; dental contact person	Referral completion; time from urgent flag to review; documented handoff rate	Shared care plan across services; oral health prompts in discharge planning; tele triage protocols; interoperable communication where feasible
**Community** **Access** **Solutions**	Proximity options (on-site/pop-up/ mobile or referral network); transport/ navigation support; affordability supports (low burden)	Outreach/service schedule; transport/navigation workflow; subsidy/enrollment steps; booking/ referral script	Outreach coordinator; navigator; community partner representative	Attendance rate; missed appointment rate; subsidy uptake; reach in high need groups	Regular community care calendar; simplified booking + reminders; culturally safe entry points (trusted venues; language access; staff continuity)
**Empowered** **Workforce** **Development**	Geriatric-focused competencies; clear task-sharing; point-of-care decision aids	Training curriculum; competency checklist; SOPs + handoffs; decision aids; supervision plan	Clinical educator; supervising dentist; aged care/home care supervisor	Training completion; competency sign-off; staff confidence/ acceptability measures	Mentorship/case conferences; onboarding + refreshers; tele-mentoring; supportive supervision model; retention supports
**Sustainable** **Service** **Models**	Task-to-skill matching; basic scheduling/referral system; minimum governance/QI cadence; supply chain plan	Operating model summary (staffing/funding/governance); procurement + maintenance SOPs; denture workflow; QI template (e.g., PDSA); contingency plan	Program manager; finance/ governance lead; procurement support	Service continuity; wait times; cost/resource-use measures; QI cycle completion	Stable financing (readiness + performance/outcome elements); procurement standardization; resilience planning; integration into existing health/aged-care governance

The examples are illustrative. Programs should adapt them to the local scope of practice, financing arrangements, and service capacity. Abbreviations: SOP = standard operating procedure; QI = quality improvement; PDSA = Plan–Do–Study–Act.

**Table 4:** Relationship of SPACES to existing frameworks and policy approaches.

Existing Approach	Main Emphasis	How SPACES Incorporates It	Added Value of SPACES
**WHO healthy aging and universal health coverage guidance**	Functional ability, dignity, equity, and access to essential services	Aligns oral health with healthy aging, function, nutrition, dignity, and universal coverage goals	Translates broad healthy aging principles into an oral-health-specific program domains for older adults
**WHO global oral health policy guidance**	Prevention, integration, workforce, surveillance, and UHC for oral health	Incorporates prevention, integration, workforce strengthening, financing, and monitoring	Provides a community program design structure rather than only high-level policy direction
**Integrated oral health and primary care models**	Screening, referral, and collaboration between dental and medical services	Includes primary care touchpoints, chronic disease reviews, closed-loop referrals, and shared information	Extends integration beyond primary care to home care, long-term care, caregivers, transport, affordability, and social support systems
**Community and mobile dental service models**	Bringing services closer to underserved populations	Includes mobile units, pop-up clinics, senior-center dental days, and on-site long-term care services	Links outreach with prevention, coordination, workforce competencies, financing, and quality monitoring
**Care coordination and** **referral-loop models**	Continuity, handoffs, and follow-up	Includes reassessment triggers, referral completion, feedback loops, and transition planning	Applies coordination principles specifically to changing oral health risks in frail, dependent, or cognitively impaired older adults
**Workforce and task-sharing frameworks**	Expanding capacity through team-based roles	Includes hygienists, dental therapists, assistants, community health workers, aged-care staff, and caregivers	Defines geriatric oral health competencies, supervision needs, escalation criteria, and role clarity for community delivery
**Sustainability and** **value-based service** **models**	Financing, governance, quality improvement, and long-term scale-up	Includes stable funding, procurement, QI cycles, outcome monitoring, and resilience planning	Connects sustainability mechanisms to oral health equity indicators and older-adult outcomes

## Data Availability

The original contributions presented in this study are included in the article. Further inquiries can be directed to the corresponding author.
